# Base Editing in Plants: Applications, Challenges, and Future Prospects

**DOI:** 10.3389/fpls.2021.664997

**Published:** 2021-07-27

**Authors:** Mawuli K. Azameti, Wadzani Palnam Dauda

**Affiliations:** ^1^National Institute for Plant Biotechnology, New Delhi, India; ^2^Indian Agricultural Research Institute, New Delhi, India; ^3^Crop Science Unit, Department of Agronomy, Federal University, Gashua, Nigeria

**Keywords:** genome editing, base editing, CRISPR/Cas9, plant, crop improvement

## Abstract

The ability to create targeted modifications in the genomes of plants using genome editing technologies has revolutionized research in crop improvement in the current dispensation of molecular biology. This technology has attracted global attention and has been employed in functional analysis studies in crop plants. Since many important agronomic traits are confirmed to be determined by single-nucleotide polymorphisms, improved crop varieties could be developed by the programmed and precise conversion of targeted single bases in the genomes of plants. One novel genome editing approach which serves for this purpose is base editing. Base editing directly makes targeted and irreversible base conversion without creating double-strand breaks (DSBs). This technology has recently gained quick acceptance and adaptation because of its precision, simplicity, and multiplex capabilities. This review focuses on generating different base-editing technologies and how efficient they are in editing nucleic acids. Emphasis is placed on the exploration and applications of these base-editing technologies to enhance crop production. The review also highlights the drawbacks and the prospects of this new technology.

## Introduction

Over the years, few genome editing technologies emerged to help to modify the genomes of plants and animals for various reasons. Notable among them is the clustered regularly interspaced short palindromic repeat (CRISPR)-CRISPR-associated protein (CRISPR/Cas) technology, which has been the most used to improve upon crops (Yu et al., 2017; Li et al., 2019).

CRISPR/Cas has gained worldwide attention as an efficient genome editing technology in plants and animals. The simplicity, versatility, and cost-effectiveness of this technology have led to a great agricultural revolution. This system uses the Cas9-sgRNA complex to create breaks in the double-strands of DNA in the organism. These double-strand breaks (DSBs) are corrected either through a non-homologous end joining (NHEJ) approach or homology-directed repair (HDR) mechanism (Danner et al., 2017). While HDR shows a high-fidelity in its repair mechanism resulting in the insertion or replacement of gene, the NHEJ is prone to errors and randomly makes indels (Voytas and Gao, 2014). HDR-mediated gene replacement has therefore been regarded as a better choice to carry out gene editing across plant species (Lee et al., 2018). However, using the HDR approach in plants is still a daunting task due to its inherently low frequency and the few numbers of donor repair templates (DRTs) delivery to the cells of plants (Sun et al., 2016; Lee et al., 2018).

**Figure 1:**
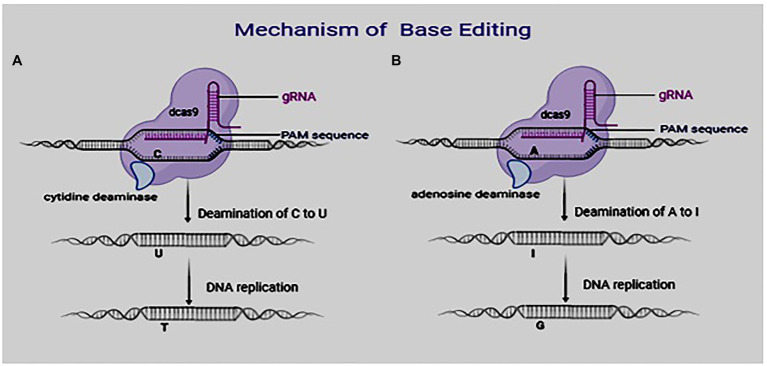
Mechanism of base editing system: **(A)** Base editing using cytosine base editors (CBEs) and **(B)** base editing using adenosine base editors.

Studies of agronomic traits revealed that many such traits are determined by single changes in the bases of genes (Li et al., 2017). Unfortunately, the CRISPR/Cas9 system cannot be used to carry out gene base conversion. They are most appropriate in knock-out or knock-in of genes. Owing to these limitations, it is imperative to look for a precise and stable approach for editing crop genomes. Base editing has been regarded as an alternative and more efficient approach (Veillet et al., 2019). It is a simple and precise approach for nucleotide conversions without the formation of DNA DSBs (Figure 1; Yin et al., 2017; Davies, 2019). It overcomes some limitations of CRISPR/Cas9 by its utilization of a tethered deaminase domain or nickase Cas9 for base conversion from A > G or C > T. Recent studies have utilized this technique to create both single and multiple nucleotide modifications in cells (Wang et al., 2020). Hence, automatic plant genetic engineering with high-throughput is possible using this technology. Although it seems to have a simplistic conceptual frame, a proper understanding of its basic concept, implementation, and possible drawbacks is essential. This current study offers a synthesis of available information concerning the aforementioned gaps in this technology and offers further possibilities in crop improvement using the base editing of plant genomes.

## Currently Available Types of Base Editors

Targeted substitution of one base or base pair with another without creating DSBs is termed as base editing. This is carried out with either DNA or RNA base editors. A base editor contains an inactive CRISPR–Cas9 component (Cas9 variants, dCas9 or Cas9 nickase) and a deaminase (cytosine or adenosine) component, which functions in converting one base to another. The change of one base to another has the potential of generating new crop varieties, thereby enhancing crop improvement processes. Two groups of base editors exist DNA and RNA base editors.

### DNA Base Editors

DNA base editors are generally made up of catalytically inactive nuclease fused to a catalytically active enzyme responsible for modifying the base. Two DNA base editors are currently in use: the cytidine base editor (CBE) and adenine base editor (ABE). There is a cytosine (C) deamination to produce uracil (U) using CBE. During DNA replication, the uracil (U) is read as thymine (T). CBE, therefore, creates C·G to T·A single-base substitution (Figure 1; Komor et al., 2016). Unlike CBE, where the inactive CRISPR–Cas9 domain is linked to a cytidine deaminase, in ABE, they are linked to adenosine deaminase, which helps to convert adenine (A) to inosine (I; Figure 1). This inosine is read as guanine (G) during DNA replication. Therefore, ABE creates A·T to G·C base substitutions (Nishida et al., 2016). Base editors have, since their discovery, become efficient tools to precisely modify genomes of eukaryotic organisms (Hua et al., 2018; Liu et al., 2018; Qin et al., 2020).

#### Cytosine Base Editors

The initial version of cytosine base editors (CBEs), known as the first-generation cytosine base editors, was created by joining cytidine deaminase (a rat apolipoprotein B mRNA editing enzyme (rAPOBEC1)) to the N terminus of dCas9 (Komor et al., 2016). They create a change of C-to-T in DNA (Rees and Liu, 2018; Ranzau and Komor, 2019) by removing the outside amine group of the target C, which leads to the generation of U. The uracil is read as thymine by the DNA polymerase during DNA replication. There is, however, a base excision repair (BER) mechanism that reverses the C·G to T·A conversion. Cellular repair systems recognize any G: U base pair as a mismatch. The BER activity removes the uracils with the help of uracil N-glycosylases (UNGs). This phenomenon renders the BE1 system inappropriate for editing single bases *in vivo* (Komor et al., 2016).

Given this limitation coupled with its low efficiency, it became essential to develop more improved versions. BE2 (APOBEC-XTEN-dcas9-UGI), a second-generation base editor, was therefore developed. In this base editor, a uracil DNA glycosylase inhibitor (UGI) was added to the DNA targeting module at the C-terminus (Komor et al., 2016). In so doing, the role of UNG that removes the uracil from DNA in cells and induces BER mechanism is inhibited. It also resulted in a 3-fold increase in editing efficiency (Komor et al., 2016). BE2 creates very few indels (<0.1%) during base editing, which makes it an appropriate choice for applications, where indels are not desirable (Komor et al., 2016).

**Figure 2:**
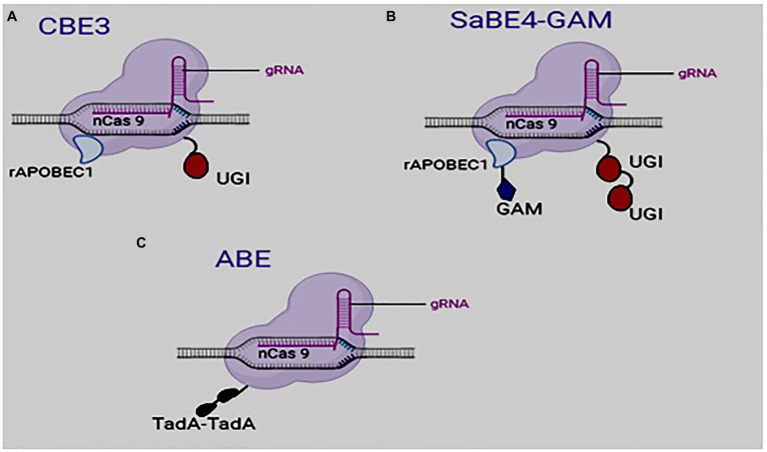
Structure of some developed base editors: **(A)** Cytosine base editor (third generation), **(B)** *Staphylococcus aureus* Cas9-derived BE4 and **(C)** adenosine base editor.

A third-generation editor, BE3, was subsequently developed by fusing rAPOBEC1 and UGI to the N and C termini of nickase cas9 D10A, respectively, (Figure 2; Komor et al., 2016). BE3 cannot cleave dsDNA but can create a cut in the non-edited strand (Komor et al., 2016). In effect, BE3 creates less off-target editing events than Cas9 (Komor et al., 2016; Kim D. et al., 2017). The use of BE3 for base editing gives a 6-fold increase in efficiency over BE2 (Mishra et al., 2020).

The quest to improve upon the efficiency of base editing led to the development of BE4 (*Streptococcus pyogenes* Cas9-derived base editor) and SaBE4 (*Staphylococcus aureus* Cas9-derived BE4), which are regarded as the fourth-generation base editors. In these base editors, the rAPOBEC1 was linked to Cas9D10A at the N terminus, while two UGI molecules were fused to the C terminal of Cas9 nickase (Komor et al., 2017). An improved version of the BE4 and the SaBE4 was created by fusing a DNA end-binding protein Gam, which is produced from bacteriophage Mu, to the Cas9 nickase at the N terminus (Figure 2). The resultant editors (BE4-GAM and SaBE4-GAM) proved to be more efficient in base editing than the BE4 and the SaBE4. The presence of the UGI minimizes the formation of unwanted by-products by blocking the UNG from getting access to the uracil intermediate, thereby preventing BER.

Even though the C-G to T-A conversion is done in a much-programmed approach, non-target conversion may occur when two or more cytosines (Cs) are situated in the catalytic window. This necessitated the engineering of several Cas9 variants (using non-canonical PAM). One of such engineered variants of BE3 is HF-BE3. The activity of dCas9 in HF-BE3 has been reduced to avoid non-specific interactions with the DNA phosphate group (Kleinstiver et al., 2016).

There are two main challenges regarding the use of BE3 editors; the need for an NGG PAM sequence (which limits the editable sites) and off-target editing. Kim Y. et al. (2017) addressed these limitations by developing several enhanced BE3 editors. They used Cas9 homolog from *S. aureus* (SaCas9; Ran et al., 2015) to engineer dCas9 variants. The SpCas9 variants (VQR-BE3, EQR-BE3, VRER-BE3, and SaKKHBE3) led to a 2.5-fold increase in editing efficiency (Kim Y. et al., 2017; Table 1). They also developed editors with altered activity windows to correct the off-target editing of BE3s.

**Table 1:** Applications of base editing in plants.

Editor	Structural features	Plant	Target genes	Gene function	Phenotype/Trait improvement	Editing efficiency (%)	Reference
BE3	pnCas9-PBE	Rice	*OsCDC48*, *OsNRT1.1B*, *OsSPL14*	*OsCDC48* regulates senescence and cell death. NRT1.1B encodes a nitrogen transporter. *OsSPL14* controls grain yield and grain number	Reduced senescence and death.	43.48	Zong et al., 2017
BE2	APOBEC1-XTEN-Cas9(D10A)	Rice	*NRT1.1B*, *SLR1*	NRT1.1B encodes a nitrogen transporter. SLR1 encodes a DELLA protein	Editing in NRT1.1B increased nitrogen use efficiency Substitution in SLR1 led to plants with semi-dwarf phenotype	≤13.3	Lu and Zhu, 2017
BE3	APOBEC1-XTEN-Cas9n-UGI-NLS	Rice	*OsCERK1*, *OsSERK1*, *OsSERK2*, *ipa1*	Encodes receptor-like kinase. *Ipa1* encodes *OsSPL14* involved in regulating rice plant architecture	Detect the efficiency of rBE3	10–38.9	Ren et al., 2017
BE4	APOBEC1-XTEN-Cas9n (VQR)-UGI-NLS	Rice	*pi-ta*	Blast susceptible protein	-	18.2	
Target-AID	nCas9^Os^-PmCDA1^At^	Rice	*ALS*	Encodes acetolactate synthase, which functions in the biosynthesis of the branched amino acid	Herbicide resistance	6–89	Shimatani et al., 2017
ABE	PABE	Rice	*OsALS*, *OsCDC48*, *OsAAT*, *OsDEP1*, *OsACC*, *OsNRT1.1B*, *OsEV*, *OsOD*	*OsALS* encodes acetolactate synthase, which functions in the biosynthesis of the branched amino acid *OsCDC48* regulates senescence and cell death.	Development of efficient ABE PABE-7	3.2–59.1	Li et al., 2018
ABE	pRABEsp-OsU6	Rice	*OsSPL14*, *OsSPL16*, *OsSPL18*	They control grain yield and grain number	High yield	>4.8	Hua et al., 2018
ABE	pRABESA-OsU6sa	Rice	*OsSPL14*, *OsSPL17*, *OsSPL16*, *OsSPL18*	They control grain yield and grain number	High yield	>17	
ABE	VQR-Cas9 (D10A)/VRER-Cas9 (D10A)	Rice	*OsSPL14*, *OsSPL17*, *OsSPL16*, *OsSPL18*	Controls grain yield and grain number	High yield	≤74.3	Hua et al., 2019a
BE3	xCas9(D10A)-rAPOBEC1	Rice	*OsDEP1*	Controls yield associated traits	-	≤30	Zhong et al., 2019
Target-AID	xCas9(D10A)-PmCDA1	Rice	*OsDEP1*	Controls yield associated traits	-	<20	
Target-AID	Cas9-NG (D10A)-PmCDA-UGI	Rice	*OsDEP1*, *OsCDC48*	*OsDEP1* controls yield associated traits *OsCDC48* regulates senescence and cell death	-	0–56.3	
Target-AID	NGv1 (D10A)	Rice	*EPSPS*, *ALS*, *DL*	Herbicide tolerance	Herbicide tolerance	5–95.5 4.2–86.3	Endo et al., 2019
BE	NGv1 (D10A)	Rice	*EPSPS*, *ALS*, *DL*	Herbicide tolerance	Herbicide tolerance	4.3–21.8	
BE3 Target-AID ABE	eBE3, eCDA, eABE	Rice	*OsACC*	Herbicide tolerance	Herbicide tolerance	–	Liu et al., 2020
BE3 ABE	Base-Editing-mediated Gene Evolution (BEMGE)	Rice	*OsALS1*, *OsALS2*, *OsALS3*	Encodes acetolactate synthase, which functions in the biosynthesis of the branched amino acid	Herbicide tolerance	–	Kuang et al., 2020
BE3 Target-AID	xCas9-epBE	Rice	*OsMPK2*, *OsMPK5*, *OsALS*, *OsNRT1.1B*	*OsALS* encodes acetolactate synthase, which functions in the biosynthesis of the branched amino acid. NRT1.1B encodes a nitrogen transporter. *OsMPK2* and *OsMPK5* encode a stress-responsive rice mitogen-activated protein kinase	-	5–64.3	Zhang et al., 2020b
BE4 ABE	xCas9n-CBE, Cas9n-NG-CBE, eCas9n-NG-CBE xCas9n-ABE, Cas9n-NG-ABE, eCas9n-NG-ABE	Rice	*OsWaxy*, *OsEUI1*, *OsCKX2*, *OsWaxy*, *OsEUI1*, *OsCKX2*	*OsWaxy* encodes starch synthase enzyme I. *OsEUI1* (*Elongated Uppermost Internode 1*). *OsCKX2* encodes a cytokinin oxidase/dehydrogenase	-	9.1–45.5 2–6.5	Zeng et al., 2020
ecTadA∗7.10-nSaCas9	ABE-P2S	Rice	*SPX-MSF2*, *OsSPL14*, *OsSPL17*, *OsSPL16*, *OsSPL18*	Control grain yield and grain number		15.9–61.1	Hua et al., 2020a
ecTadA∗7.10-nSaKKH-Cas9	ABE-P5S	Rice	*OsSPL13*, *SNB*	*OsSPL13* controls grain yield and grain number *SNB* regulates the transition of spikelet meristems into floral meristems in rice	-	6.1–33.9	
ABE	ABE7.10-nSpCas9-NGv1	Rice	*sgOs-siteG1*, *sgOs-site2*, *sgOs-site3*, *sgOs-site4*	-	-	29.2–45.8	Negishi et al., 2019
PE	Sp-PE2, Sp-PE3, Sa-PE3	Rice	*ALS*, *APO1*, *SLR1*, *OsSPL14*, *APO2*	*ALS* encodes acetolactate synthase, which functions in the biosynthesis of the branched amino acid. SLR1 encodes a DELLA protein. *OsSPL14* controls grain yield and grain number. APO1 and APO2 are positive regulators of panicle size and grain number.	-	0–17.1	Hua et al., 2020b
PE	pPE2, pPE3, pPE3b	Rice	*OsPDS*, *OsACC1*, *OsWx*	*OsACC1* plays a role in herbicide tolerance *OsPDS* is involved in nutritional improvement.	-	0–31.3	Xu et al., 2020b
PE	PE-P1, PE-P2	Rice	*OsALS*, *OsACC*, *OsDEP1*	Herbicide tolerance	Herbicide tolerance	≤26	Xu et al., 2020c
STEME	APOBEC3A–ecTadA–ecTadA* nCas9 (D10A)	Rice	*OsACC*	Herbicide tolerance	Herbicide tolerance	15.10	Li et al., 2020a
PE	pCXUN-Ubi-NLS-nCas9(H840A)-Linker1 (33aa)-M-MLV-RT-Linker2 (14aa)-NLS-PolyA-E9-Actin-Nos	Rice	*hptll*, *OsEPSPS*	*HptII* confers hygromycin resistance. *OsEPSPS* gene encodes a key enzyme in the synthesis of aromatic amino acids	-	2.22–9.38	Li et al., 2020c
APOBEC3A	A3A-PBE	Wheat	*TaALS*, *TaMTL*, *TaLOX2*	*TaALS* encodes acetolactate synthase, which functions in the biosynthesis of the branched amino acid. *TaLOX2* encodes a lipoxygenase enzyme involved in the hydrolysis of polyunsaturated fatty acids	-	16.7–22.5	Zong et al., 2018
BE3	PBE	Wheat	*TaALS-P174*	Encodes acetolactate synthase, which functions in the biosynthesis of the branched amino acid	Herbicide resistance	33–75	Zhang et al., 2019
Target-AID	pDeSpnCas9-NG_PmCDA1_UGI	Tomato	*ALS*	Encodes acetolactate synthase, which functions in the biosynthesis of the branched amino acid	Herbicide resistance	32	Veillet et al., 2020b
BE3	CBE3	Watermelon	*ALS*	Encodes acetolactate synthase, which functions in the biosynthesis of the branched amino acid	Herbicide resistance	23	Tian et al., 2018
BE3	GhBE3	Cotton	*GhCLA*, *GhPEBP*	*GhCLA* functions in chloroplast development. *GhPEBP* participates in the multiplex‐branch developmental process	-	26–58	Qin et al., 2020
BE3	pTF101.1-sgRNA-BE	Soybean	*GmFT2a*, *GmFT4*	Function in flowering induction in soya bean.	-	≤18.2	Cai et al., 2020
BE3	CBE	Oilseed rape	*BnALS1*	Herbicide tolerance	Herbicide tolerance	1.8	Wu et al., 2020

-, not reported.

#### Adenine Base Editors

Adenine base editors (ABEs) are very similar to CBEs in both structure- and base-editing mechanisms. However, ABEs possess an adenosine deaminase instead of the cytidine deaminase found in the CBEs.

ABE is made up of three main parts: a mutant transfer RNA adenosine deaminase (TadA), a sgRNA, and a Cas9 nickase (Figure 2). Like cytosine, an adenine can be deaminated to produce inosine (Figure 1), which is read as G and paired with C during DNA replication.

The earliest version of ABEs (ABE1.2) was developed by joining the N-terminus of the nCas9 to the TadA- TadA* heterodimer by the use of the 16-amino-acid linker XTEN, while the C terminal of nCas9 was linked to a nuclear localization signal (NLS). Over time, various improvements and optimizations were carried out on ABE to enhance editing efficiency. The use of different TadA mutations and linking the TadA (2.1)* domain to the C-terminus of nCas9 (D10A), employing varying length of the linker between TadA (2.1)* and nCas9 (D10A), or the use of an inactivated N-terminal TadA* subunits are some of the strategies adopted to improve ABE editing efficiency.

Seventh-generation ABEs were developed through thorough directed evolution and protein engineering. These ABEs, such as ABE7.10, can achieve up to 50% efficiency in human cells, with higher product purity (mostly ≥99.9%) and very few indels (typically ≤0.1%; Gaudelli et al., 2017). ABE7.10 contains 14 amino acid substituted in the catalytic TadA* domain, and it has the highest efficiency to date. Its editing window is approximately 4–7 protospacer positions, counting the PAM as positions 21–23 (Gaudelli et al., 2017). Three other evolutionary related constructs ABE 6.3, ABE 7.8, and ABE 7.9 have slightly larger editing windows at the expense of their editing efficiency (Gaudelli et al., 2017). Off-target activity by ABE7.10, ABE7.9, and ABE 7.8 in HEK 293 T cells appeared to be lower than that caused by standard Cas9 editing, and no ABE-induced editing was detected outside on-target or off-target protospacers. The editing efficiency of the ABE7.10 was subsequently improved by adding modified NLS and codon-optimization (ABEmax; Koblan et al., 2018).

A more recent simplified base editor ABE-P1S (Adenine Base Editor-Plant version 1 Simplified), which contains ecTadA*7.10-nSpCas9 (D10A), exhibited improved efficiency in editing in rice as compared to the widely used ecTadA-ecTadA*7.10-nSpCas9 (D10A) fusion (Hua et al., 2020a).

ABE8 variants (i.e., TadA8e, TadA8.17, and TadA8.20) have recently been generated by the introduction of more mutations into eTadA* (Gaudelli et al., 2020; Lapinaite et al., 2020; Richter et al., 2020). These exhibited increased editing efficiencies in human cells. Out of these editors, TadA8e has the highest efficiency of creating A-to-G changes across different target sites (Yan et al., 2021). Yan et al. (2021) developed an improved base editor, named as TadA9, by introducing V82S/Q154R mutations in TadA8e. TadA9 was determined to be very compatible with various nickase systems, such as CRISPR/SpCas9, CRISPR/SpCas9-NG, CRISPR/SpRY, and CRISPR/ScCas9, and it exhibited high efficiency in editing four herbicide target genes in commercial rice.

The engineering of ABEs was necessitated as a result of the rapid progress made in the development of CBEs.

### Dual Base Editors

Currently, single-function base editors (CBE and ABE) are capable of creating only transition base changes; C·G→T·A and A·T→G·C. This limits the scope of editing patterns that these base editors can create within a particular target site. Scientists thought of creating base editors possessing the ability to create both C→T and A→G base substitution, which would expand the potential of base editing. To create these new dual base editors, attempts were made to attach both deaminases; the cytidine and the adenosine deaminase, to Cas9. This is directed to the targeted stretch of DNA using an easily programmable guide RNA.

The first invention of a dual base editor was reported by a group of scientists led by Caixia Gao (Li et al., 2020a). They engineered a dual base editor named as “saturated targeted endogenous mutagenesis editors” (STEMEs) that is capable of simultaneously performing C: G > T: A and A: T > G: C editing in plants by the use of a single sgRNA. STEME consists of a fusion of cytidine deaminase (APOBEC3A), an adenosine deaminase (ecTadA–ecTadA*), nCas9 (D10A), and a UGI fusion. The STEME system carries out the deamination of cytidines and adenosines to uridine and inosines, respectively. These changes are then copied during DNA replication to generate dual C: G > T: A and A: T > G:C changes.

Following this first report, three other groups of researchers have also described the creation of their own dual base editors. Zhang et al. (2020a) reported the creation of A&C-BEmax. This was created when both deaminases were fused with a Cas9 nickase to generate simultaneous C-to-T and A-to-G substitutions within the targeted site. Unlike the single base editors, A&C-BEmax have reduced editing efficiency on adenines while its activity on cytosines is enhanced (Zhang et al., 2020a).

Sakata et al. (2020) developed three dual base editors, Target-ACE, Target-ACEmax, and ACBEmax, with both cytidine and adenosine deaminases linked to a single nCas9 (D10A).

In Target-ACE, the nCas9 was fused to PmCDA1 from Target-AID at the C-terminus while the TadA heterodimer from ABE7.10 was fused to the Nterminus. The Target-ACE was codon-optimized and N-terminal bipartite NLS was added to develop Target-ACEmax. In ACBEmax the codon-optimized PmCDA1 domain of Target-ACEmax was replaced with the codon-optimized cytidine deaminase domain rAPOBEC1 from BE4max.

Grünewald et al. (2020) developed a dual base editor named as “synchronous programmable adenine and cytosine editor” (SPACE) that is capable of simultaneously creating A-to-G and C-to-T substitutions, hence broadening the scope of possible DNA sequence alterations. SPACE was developed by combining Target-AID (a CDA1-based CBE) and adenosine deaminase from miniABEmax-V82G. The researchers added two UGIs in order to enhance the purity of on-target cytosine base edits created by SPACE.

Li et al. (2020a) used STEME editors in rice to produce herbicide resistant rice. In rice protoplasts, STEME-1 generated 15.10% editing efficiency of C > T and A > G. STEME-1 and STEME-NG were applied to edit rice gene OsACC, leading to the creation of herbicide resistance mutations (Li et al., 2020a).

Kuang et al. (2020) developed a base-editing-mediated gene evolution (BEMGE) method, which employs the combination of both Cas9n-based cytosine and adenine base editors in addition to a single-guide RNA (sgRNA). The researchers used the BEMGE method to artificially evolve OsALS1 in rice cells. They derived four different amino acid substitutions from two novel sites in the OsALS1, conferring different levels of tolerance to the herbicide bispyribac-sodium.

### Transversion Base Editors

Even though both CBEs and ABEs were employed in various organisms to induce single base changes, their application is limited. This is because they can only induce base transition. This implies that CBE and ABE can achieve only four (33.3%) out of the total number of 12 possible base substitutions.

Recently, different groups of researchers sought to create transversion base editors to enhance the applicability of base editing.

C-to-G Base Editors (CGBE) are created by fusing a Cas9 nickase (nCas9-D10A) to a cytidine deaminase and a UNG. The cytidine deaminase, guided by RNA, induces the conversion of a target C to U. The UNG detects and removes the U from the DNA, thereby creating an apurinic/apyrimidinic (AP) site. The creation of the AP site and nicking at the non-edited strand by nCas9 elicits DNA repair and replication mechanisms leading to the insertion of G at the AP site. Interestingly, the mechanism by which G is chosen over the other two bases remains unclear. The presence of UNG in CGBE differentiates them from CBEs, which contain UNG inhibitor (UGI).

Recently, three groups of scientists have independently created cytosine transversion base editors (Chen et al., 2020; Kurt et al., 2020; Zhao et al., 2020). Kurt et al. (2020) engineered two base editors (CGBE1 and miniCGBE1) capable of efficiently inducing targeted C-to-G changes with an editing efficiency of 71.5%. CGBE1, which is made up of RNA-guided Cas9 nickase, a uracil DNA N-glycosylase (eUNG) derived from *Escherichia coli*, and a rat APOBEC1 cytidine deaminase variant (R33A), was reported to efficiently create C-to-G changes, especially in AT-rich sequences. The removal of eUNG domain from the CGBE1 to create miniCGBE1 led to a reduction in indel frequencies and editing efficiency (Kurt et al., 2020). CGBE1 and miniCGBE1 have the potential of forming the basis for optimizing C-to-G base editors for crop improvement. Zhao et al. (2020) developed BEs called as glycosylase base editors (GBEs), which could induce C-to-A and C-to-G changes. The GBEs developed consists of complexes of fused nCas9, activation-induced cytidine deaminase (AID), and uracil-DNA glycosylase (Ung). Chen et al. (2020) developed C: G to G:C Base Editors (CGBEs) capable of creating base transversions in human cells with an editing efficiency of 15 ± 7%. The CGBEs were developed by fusing a nickase CRISPR-Cas9 (nCas9) to a cytosine deaminase and XRCC1, a BER protein. The function of the XRCC1 is to recruit other BER proteins to repair the AP site, which results in G as the predominant product (Chen et al., 2020).

### PAMless Base Editors

The purposeful engineering of CRISPR enzymes capable of targeting previously inaccessible PAMs is one way of expanding the scope of genome editing technologies. SpCas9 ordinarily recognizes NGG PAM.

Different groups employed various approaches to independently reduce the 5ꞌ-NGG-3ꞌ PAM specificity of SpCas9 to a single guanine (G) nucleotide, either through phage-assisted continuous evolution (xCas9-3.7; Hu et al., 2018), structure-guided rational design (SpCas9-NG; Nishimasu et al., 2018), or bioinformatics approach (*Streptococcus canis* Cas9; ScCas9; Chatterjee et al., 2018).

Although these variants expand SpCas9’s potential targeting space, they possess some drawbacks. For instance, SpCas9-NG exhibits less editing efficiency on 5ꞌ-NGC-3ꞌ PAM targets while demonstrating increased level of off-target (Nishimasu et al., 2018). ScCas9 has reduced editing efficiencies within various gene contexts (Chatterjee et al., 2018), while xCas9-3.7 possesses higher fidelity instead of broad PAM recognition (Hua et al., 2019a; Zhong et al., 2019).

There is, therefore, the need to develop Cas9 enzyme that combines broad genomic accessibility with high editing efficiency and specificity. Walton et al. (2020) using guided engineering strategies, developed variants of *Streptococcus pyogenes* Cas9 (SpCas9). This variant named as SpG can recognize a wide range of NGN PAMs. The researchers further optimized SpG to develop SpRY that has the potential of targeting almost all PAM. Xu et al. (2020a) have researched the effectiveness of both SpG and SpRY nucleases against separate PAMs and their use in both cytosine and adenine base editing using transgenic rice callus. They reported that even though SpG recognizes NG PAM sequences, its performance is less when compared to SpCas9-NG in rice. However, SpRY has a higher performance than SpCas9-NG but has the tendency of exhibiting self-targeting activity on transfer T-DNA sequence. These could be employed to facilitate genome editing in plants and to expand the scope of applications in agriculture and plant biology.

In separate research, Mok et al. (2020) identified a bacterial toxin named as DddA, which induces the deamination of cytidines in double-stranded DNA. They developed split DddA halves that were non-toxic and inactive until the programmable DNA-binding proteins adjacently linked to the target DNA were brought together. It was determined that the fusion of these split halves with transcription activator-like effector array proteins and UGI produced RNA-free DddA-derived cytosine base editors (DdCBEs) that caused C-G to T-A editing in human mtDNA. Chatterjee et al. (2020) developed an optimized ScCas9 enzyme by the use of evolutionary knowledge from closely related orthologs to create two modifications to the original open reading frame (ORF). This generated Sc^++^ with broad editing potentials. Finally, they engineered a high-fidelity variant of Sc^++^ (HiFi-Sc^++^) with an enhanced specificity, while maintaining the on-target efficiency or accessibility.

## Multiplex Base Editing

Various traits of agronomic importance in plants are controlled by single-nucleotide substitutions (Chen et al., 2019). It is therefore expected that stacking traits or altering various major factors of regulatory pathways would greatly enhance crop improvement (Chen et al., 2019). This can be mostly achieved through multiplexed genome editing.

To perform multiplex editing, researchers mostly use the number of orthogonal CRISPR systems which form the multi-functional CRISPR system, including SpCas9 and SaCas9 variants, which perform dual functions. Tri-functional method, such as LbCpf1 variant for CRISPRa, SpCas9 variant for CRISPRi, and SaCas9 variant for deletion, are also mostly used (Lian et al., 2017). One drawback of these techniques is that they involve delivering several Cas proteins simultaneously with each Cas protein requiring its own PAM sequence (Lian et al., 2017).

Recently, a group of scientists developed a CRISPR-based system named as “simultaneous and wide editing induced by a single system” (SWISS), which is capable of inducing multiplexed and simultaneous base editing in rice (Li et al., 2020b). SWISS works on the principle that RNA aptamers enlist their own cognate binding proteins in the engineered scRNAs fused with both deaminases. This helps to generate simultaneous CBE and ABE edits at the target sites. The use of another pair of sgRNAs in addition to this dual-function system generates a DSB at a third target site, thereby creating a tri-functional genome editing at multiple sites.

## Prime Editing

Prior to the development of transversion base editors, a ground-breaking genome editing technology was developed that addresses the issue of transversion editing. This technology, known as the “prime editor,” will create 12 kinds of base substitutions in human cells. The prime editor is made up of nCas9 (H840A) fused with Moloney murine leukemia virus (M-MLV RT) reverse transcriptase, which a prime editing guide RNA (pegRNA) consisting a reverse transcriptase template and a primer-binding site at the 3ꞌ end of the sgRNA. The genetic information for the target mutation is contained in the reverse transcriptase template while the primer-binding site connects the nCas9 (H840A)-nicked ssDNA strand. This primes the reverse transcription and incorporates the genetic information from the reverse transcriptase template into the genome. The 3ꞌ extension of the pegRNA containing both a primer binding site (PBS) and a reverse transcription sequence (RT sequence) enables the insertion of the desired polymorphism at the target site after the ssDNA cuts around 3-bp upstream of the PAM sequence on the nontarget strand (Anzalone et al., 2019). Even though the prime editor produces base replacements and brief indels at a comparatively broad range of positions (+1 to+33), it is not limited by its PAM.

This system was developed and used in few crops including rice, wheat, and maize (Hua et al., 2020b; Lin et al., 2020; Xu et al., 2020b; Table 1). Lin et al. (2020) applied prime editing technology in rice and wheat and recorded point mutations, insertions, and deletions in the protoplasts of the plants. They obtained regenerated prime-edited rice plants at frequencies of up to 21.8%. Xu et al. (2020b) developed and tested the activity of a plant prime editor 2 (pPE2) on an HPT^-ATG^ reporter in rice. They reported editing frequency of up to 31.3% in transgenic T_0_ plants. Xu et al. (2020c) used prime editor to target OsALS-1 and OsALS-2 and recorded the expected G-to-T and specific C-to-T substitutions editing efficiencies of 1.1% (1/87) and 1.1% (1/88), respectively. Hua et al. (2020b) developed a prime editor Sp-PE3 and tested its efficiency in rice calli and recorded an editing efficiency of up to 17.1% at the targeted sites. The developed prime editor Sp-PE3 was used to edit the rice endogenous acetolactate synthase (ALS) gene, leading to a desired G-A base transition in 4 out of 44 (9.1%) transgenic lines, with no insertions or deletions. Interestingly, no mutation was found at ABERRANT PANICLE ORGANIZATION 1 (APO1) site using the same editor, suggesting that Sp-PE3 can induce precise base substitution with varied efficiency depending on the targeted site.

Jiang et al. (2020) used prime editors to generate mutant maize lines harboring W542L/S621I double mutations. Using prime editor pZ1WS developed, they observed 43.75% (7 of 16) lines transformed with pZ1WS harbored the S621I edit, with one line displaying homozygous mutations in both ZmALS1 and ZmALS2. Similarly, Butt et al. (2020) attempted to engineer herbicide resistance by targeting rice ACETOLACTATE SYNTHASE (OsALS) and recorded editing efficiency of 0.26–2% was recorded at the targeted site. The researchers engineered herbicide resistance trait in rice through base substitutions. Besides, Veillet et al. (2020a) reported a successful targeting of potato *StALS* genes using CRISPR-mediated plant prime editing (PPE) and recorded 92% editing efficiency.

Despite the use of orthogonal techniques, including reverse transcriptase orthologs having varied catalytic activities, the efficiency of editing using the prime editors remains limited in plants (Lin et al., 2020; Xu et al., 2020c).

## Applications of Base Editors in Crop Improvement

Several reports indicated that CBEs and ABEs are successfully used in editing specific genes conferred by single changes in bases in various crops (Table 1).

### Application of CBEs in Crop Improvement

Since the first successful establishment of the CBE system in plants (Li et al., 2017; Lu and Zhu, 2017), they have been employed in various studies to improve traits in crops (Table 1). Two rice genes (NRT1.1B and SLR1) were reported to possess some agricultural importance (Lu and Zhu, 2017). While the NRT1.1B gene codes for a nitrogen transporter, the SLR1 gene codes for a DELLA protein.

Initial studies indicate that a C to T substitution in NRT1.1B enhanced nitrogen use efficiency in rice (Hu et al., 2015). A point mutation was therefore induced in these genes (NRT1.1B and SLR1) using the CRISPR/Cas9-xyr5APOBEC1 based editing system. The researchers achieved a substitution of C with T at a frequency of 1.4–11.5%, resulting in improved nitrogen use efficiency (Hu et al., 2015). Another successful application of CBE in crop improvement was recorded when the *acetolactate synthase* (ALS) gene was edited in tomatoes using CBEs, resulting in enhanced resistance to herbicide in tomatoes (Shimatani et al., 2017).

Transformation of a maize-codon-optimized cytidine deaminase-Cas9n-UGI (CBE3) into *Arabidopsis* led to 1.7% C·G to T·A mutation efficiency in Pro197 of the ALS gene, resulting in herbicide resistance in the T_2_ generation (Chen et al., 2017). In rice, *Pi-d2* and *OsFLS2* genes were successfully edited by rBE5 at 30.8 and 57.0% editing efficiencies respectively, resulting in improved blast tolerance in the edited plants (Ren et al., 2018).

Rice base editors (rBE3 and rBE4) were used to create changes in the genetic make-up of rice (Ren et al., 2017). In the study, the expression of base editor rBE3 in the rice leaf sheath protoplasts along with OsCERK1-targeting sgRNA, and its subsequent optimization using human AID (hAID) mutant version (hAID*Δ) led to an improvement in the efficiency of base editing (Ren et al., 2018). Shimatani et al. (2017) reported the introduction of multiple point mutations responsible for herbicide resistance in rice plants by employing multiple base-editing approaches. Similar reports indicate that point mutation in the ALS gene leads to the development of plants with increased resistance to herbicides (Yu and Powles, 2014). A3A-PBE, which has recently been engineered by using human APOBEC3A linked to Cas9 nickase, has been reported to improve upon the efficiency of base editing in plants (Zong et al., 2018).

The development of transgene-free watermelon (*Citrullus lanatus*) varieties resistant to herbicide was made possible by using a base-editing approach (Tian et al., 2018). Earlier reports indicated that single-base substitutions at various conserved positions of ALS genes help in conferring resistance to herbicides in many plants (Yu and Powles, 2014). Similarly, editing of the ALS and acetyl-coenzyme A carboxylase genes have been reported to generate wheat with enhanced tolerance to herbicides (Zhang et al., 2019). The stackable herbicide tolerance traits are regarded as useful for weed management. In another study, the researchers concluded that CBE has the potential of creating *Arabidopsis* crops resistant to imidazolinone herbicide, and the same can be used in other crops to enhance weed control (Dong et al., 2020).

In tomato, editing of the *ALS* gene using CBE led to a successful base editing at an efficiency of 71%, resulting in chlorsulfuron-resistant tomato (Veillet et al., 2019). CBE was also recently employed in Cotton (*Gossypium hirsutum*). In a study conducted by Qin et al. (2020), the researchers developed a new *G. hirsutum*-Base Editor 3 (GhBE3) base-editing approach, which was used to edit GhCLA and GhPEBP genes in cotton (*G. hirsutum*). They recorded 26.67–57.78% editing efficiency at the three target sites, which were passed on to the T_1_ progeny.

Wu et al. (2020) reported precise editing of *BnALS1* gene at position P197 in oilseed rape using CBE system. Editing efficiency of 1.8% was recorded, and the P197S substitution in *BnALS1* generated a novel herbicide-resistant mutant in oilseed rape.

### Application of ABEs in Crop Improvement

An efficient ABE was developed by Gaudelli et al. (2017), through intensive directed evolution and protein engineering. An improved ABE was used to edit the acetyl-coenzyme A carboxylase (*ACC*) gene in rice, which helped to confer herbicide resistance (Li et al., 2018; Table 1). Herbicide-resistant gene base editing enables the regeneration of edited but transgene-free plants on herbicide selection medium while simultaneously introducing additional traits through multiplexing (Zhang et al., 2019).

Liu et al. (2021) precisely edited the α-tubulin homolog gene OsTubA2 (LOC_Os11g14220) in rice using adenine base editor rBE14. They recorded 12.7% efficiency and generated novel artificial rice germplasm resistant to trifluralin and pendimethalin.

Hua et al. (2020a) demonstrated that ABE-P1S (Adenine Base Editor-Plant version 1 Simplified), which contains TadA*7.10-nSpCas9 (D10A) fusion, is more efficient in editing rice than the widely used ABE-P1, which contains TadA-ecTadA*7.10- nSpCas9 (D10A) fusion. Trait improvement in crops can therefore be made possible by adopting these more efficient ABEs. Kang et al. (2018) successfully applied plant-compatible ABE systems in *Arabidopsis thaliana* and *Brassica napus*. In this study, a single amino acid substitution in the FT protein produced plants with late-flowering while mis-splicing of the PDS3 RNA transcript resulted in plants with albino phenotypes.

In rice, Yan et al. (2018) developed a fluorescence-tracking A to G base editor (rBE14), which was used to successfully create A·T to G·C conversion in OsMPK6, OsSERK2, and OsWRKY45 in rice. This base editor is expected to help in creating DNA variations in rice for its improvement.

Recently, a novel ABEs was developed using a Cas9 variant SpCas9-NGv1. This was efficiently used to induce A to G base changes in rice (Negishi et al., 2019).

## Prospects

CRISPR–Cas has revolutionized plant research, and the use of CRISPR–Cas-derived editors helps in performing precise genome manipulations. This technology has been extensively used to improve upon the agronomic importance of various crops. Even though various improvements have been made to this editing technology to improve upon its efficiency, they still lack few needs to meet all the requirements for plant genome manipulation. There is, therefore, the need to carry out more improvement on this editing technology. One area of concern is the issue of an improved delivery system. The key to reducing obstacles to the affordable implementation of gene editing in plants would be to enhance the current delivery mechanisms and build new systems. The use of sperm cells, egg cells, and zygotes are getting attention as the realistic targets of delivery. Using pollen-mediated transformation, for example, will avoid the restrictions of species specificity and reproduction by pollination or artificial hybridization. Other promising delivery systems are those based on nanotechnology and virus particle-like structures. Carbon nanotubes, for example, have been used to transmit DNA to mature plant leaves, contributing to efficient protein expression (Demirer et al., 2018). Other nanomaterials, such as layered double hydroxides (Mitter et al., 2017), mesoporous silica nanoparticles (Cunningham et al., 2018), and polyethylenimine (Cunningham et al., 2018), also have tremendous potential to increase the supply of delivery vehicles as they can do little cellular disruption, have low toxicity, and achieve high efficiencies in transformation.

Apart from the already discussed areas, in which base editing has been applied, attention may also be given to editing mitochondrial and chloroplast genomes. Base editing could be applied in identifying cell lineages in order to understand the patterns underlying plant growth, creating genetic circuits to combine and transduce signals, and establishing plant biosensors to detect internal and external signals.

One other area, where base editing can be applied, is to accelerate the wild-plant domestication. Modern crops have been selectively bred for several years, leading to the introduction of important features, which allow high-quality, nutrient-rich food to be harvested mechanically (Østerberg et al., 2017). Major domestication traits are usually controlled by mutations in the so-called domestication genes. Base editing can be applied in such areas to enhance domestication of wild plants.

Most important agronomic traits in plants are controlled by multiple quantitative trait loci as such; editing individual genes may fail to create the necessary phenotypic change. It would therefore be imperative to create more efficient base-editing technologies capable of combining or “stacking” mutated alleles.

The application of prime editors has only been demonstrated in few plants. Its operation and efficiency in other plants also needs to be checked. In addition, the ability of the prime editor to generate greater genetic modifications (100 of bases), and its precision has not been shown. Further work is therefore required to strengthen and extend the technology for enhanced base editing in plants.

Although DNA base editors have gained prominence in the improvement of agricultural crops, a much greater precision is possible with RNA base editing (“RESCUE” and “REPAIR”) technologies. It is therefore recommended that in the future, researchers pay attention to this system for its applications in crop plants.

The application of prime editing in comparison to ABEs and CBEs offers more precise point mutation and broad applications. Hence, prime editing, which has transversion mutations ability, should also be considered an efficient alternative to single base edits. Although base editing has great potential for the improvement of agricultural plants and the development of crops of high agronomic value, its future application in an *in vivo* condition still needs great improvements.

## Conclusion

Base editing is a new technology that provides the opportunity to efficiently and precisely converting one base to another in the genome of plants and animals. It works in a programmable manner without creating DSB. This has successfully been applied in both plants and animals in the past couple of years. Its unique edge over other genome editing technologies is its non-generation of DSB, the effect on both dividing and non-dividing cells, and high precision.

However, more improvement on the base editors is recommended to optimize and enhance the scope and efficiency of editing. This includes optimizing the technology to overcome off-target effects and bystander mutation generation. Furthermore, proper sgRNAs designing employing artificial intelligence-based algorithms are other measures of overcoming some of these constraints. That notwithstanding, base editing can be used to make precise modifications in crops for sustainable production amid the current global changes.

## Author Contributions

MA conceptualized and designed the review. MA and WD wrote the paper. All authors contributed to the article and approved the submitted version.

## Conflict of Interest

The authors declare that the research was conducted in the absence of any commercial or financial relationships that could be construed as a potential conflict of interest.

## Publisher’s Note

All claims expressed in this article are solely those of the authors and do not necessarily represent those of their affiliated organizations, or those of the publisher, the editors and the reviewers. Any product that may be evaluated in this article, or claim that may be made by its manufacturer, is not guaranteed or endorsed by the publisher.
